# A modeling study of cool surfaces and outdoor workers productivity at San Francisco International Airport

**DOI:** 10.1093/pnasnexus/pgae593

**Published:** 2025-01-03

**Authors:** Barrak Alahmad, Iona Isachsen, Yazan Alwadi, Haider Taha, Anthony Bernheim, Erin Cooke, Elizabeth Wesley, Gregory Kats, John D Spengler

**Affiliations:** Environmental Health Department, Harvard T.H. Chan School of Public Health, Boston, MA 02115, USA; Smart Surfaces Coalition (SSC), Washington, DC 20009, USA; Environmental Health Department, Harvard T.H. Chan School of Public Health, Boston, MA 02115, USA; Altostratus Inc., Martinez, CA 94553, USA; San Francisco International Airport, San Francisco, CA 94128, USA; San Francisco International Airport, San Francisco, CA 94128, USA; World Resources Institute (WRI), Washington, DC 20002, USA; Smart Surfaces Coalition (SSC), Washington, DC 20009, USA; Environmental Health Department, Harvard T.H. Chan School of Public Health, Boston, MA 02115, USA

**Keywords:** climate change, smart surfaces, heat, Bay Area, albedo

## Abstract

Heat exposure in outdoor work environments poses risks to worker health and productivity. Engineering solutions like cool surfaces that increase surface albedo and reduce temperatures may help mitigate these impacts. We conducted detailed micrometeorological modeling to analyze surface characteristics and heat exposure for outdoor workers at San Francisco International Airport (SFO) under current conditions and three hypothetical albedo-increase scenarios. Wet bulb globe temperature (WBGT) was used to estimate potential productivity loss based on established relationships between heat stress and loss in physical work capacity. For the month of August 2020, we quantified possible gained hours of productivity per worker per month under each hypothetical albedo-increase scenario. Across the entire area of SFO, the average campus albedo was 0.20 (range: 0.08–0.85). Adopting low, moderate, and high albedo modifications for SFO would reduce peak midday WBGT by 0.89, 1.25, and 1.59 °C, respectively. The largest temperature reductions occurred during the morning shift (7 AM–3 PM). In one shift, we found a potential of 5.20, 7.16, and 8.95 h gained per worker over the entire month in the low, moderate, and high albedo modification scenarios, respectively.

**Significance Statement**

Even modest increases in surface albedo through cool roofs and ground surfaces have measurable potential to reduce heat exposure for outdoor workers, as demonstrated by our detailed micrometeorological modeling at a busy airport. This study quantifies potential gains in working hours under various albedo scenarios. The study provides a data-driven foundation for designing climate-resilient infrastructure in high-risk environments like airports.

## Background

Hot temperatures in the workplace that elevate the workers’ body temperature can lead to increased absenteeism (1), higher risk of workplace injuries (2), and higher incidence of acute and chronic heat-related illnesses (3). Heat is also found to reduce productivity as workers struggle with physical discomfort and fatigue when their body temperature is elevated. Productivity, defined as the physical work capacity, is the maximum physical output that can be reasonably expected from an individual performing moderate to heavy manual labor work over an entire shift (4). When heat reduces physical work capacity, it directly impacts an individual’s ability to maintain expected work output. For employers, decreased productivity due to heat can lead to substantial economic losses (5).

The International Labor Organization (6) estimated in their 2024 report that every year, at least 2.41 billion workers are exposed to excessive heat globally, with significant impacts on their health and productivity. By 2030, 2.2% of total working hours worldwide could be lost to high temperatures—a productivity loss equivalent to 80 million full-time jobs (7). In California alone, Park et al. (8) found that even a modest increase in workplace temperatures could lead to 20,000 additional injuries per year with a social cost of US$1 billion.

Mitigating heat exposure at outdoor work sites through administrative measures, such as work–rest schedules, can be challenging for employers where physical labor is essential, especially in the absence of federal standards from regulatory bodies like the US Occupational Safety and Health Administration (OSHA). However, some states, like California, have implemented their own regulations. For instance, Cal/OSHA enforces a rule (T8 CCR 3395) which mandates specific heat illness prevention measures for outdoor workplaces, including access to shade, water, and mandatory rest breaks when temperatures reach a certain threshold (9). While compliance with such standards helps to reduce heat stress, design and engineering solutions, such as implementing reflective surfaces (e.g. cool pavements and roofs), offer additional ways to lower hazardous heat exposure for workers. These cool surfaces mitigate urban overheating by increasing albedo—high albedo surfaces reflect more incoming solar radiation, decreasing the amount of energy input into the urban system, and lowering surface and air temperatures at 2-m above ground level (10). While cool surfaces have been widely adopted to address urban heat (11–13), little is known about their potential benefits for outdoor workers engaged in medium to heavy physical work.

Airports, in particular, have expansive surfaces of concrete and asphalt that may be especially prone to extreme heat. Previous studies in Saudi Arabia (14) and Thailand (15) showed that airport workers are exposed to high heat dissipated from operating vehicles, equipment in service, and the tarmac itself. Recent media reports in the United States (16, 17) are showing similar concerns where airport workers have increasingly called for greater protections. In collaboration with San Francisco International Airport (SFO), we analyzed the entire surface area of the airport and its effect on heat exposure for outdoor workers. We modeled the potential productivity loss of SFO outdoor workers under hot conditions in a “do nothing” scenario and compared it with three other scenarios involving changes in surface albedo. In each hypothetical scenario, we calculated the potential productivity gains for all shifts of SFO outdoor workers.

## Methods

### Outdoor work at SFO

SFO is located 13 miles south of downtown San Francisco, California. The airport has four terminals and multiple runways. It has an area of ∼3.54 square miles, making it the eighth largest airport in the United States, and the largest in Northern California. SFO serves as a significant hub for both domestic and international flights, handling more than 55 million passengers annually.

SFO and its airlines rely on thousands of outdoor workers to keep operations and aircraft running smoothly. Outdoor workers perform tasks such as aircraft fueling, baggage handling, marshaling, landscaping, cargo management, fire protection, security, and maintenance cleaning. In broad terms at SFO, there are three main shifts for outdoor workers: 7 AM to 3 PM; 3 PM to 11 PM; and 11 PM to 7 AM.

**Fig. 1.**
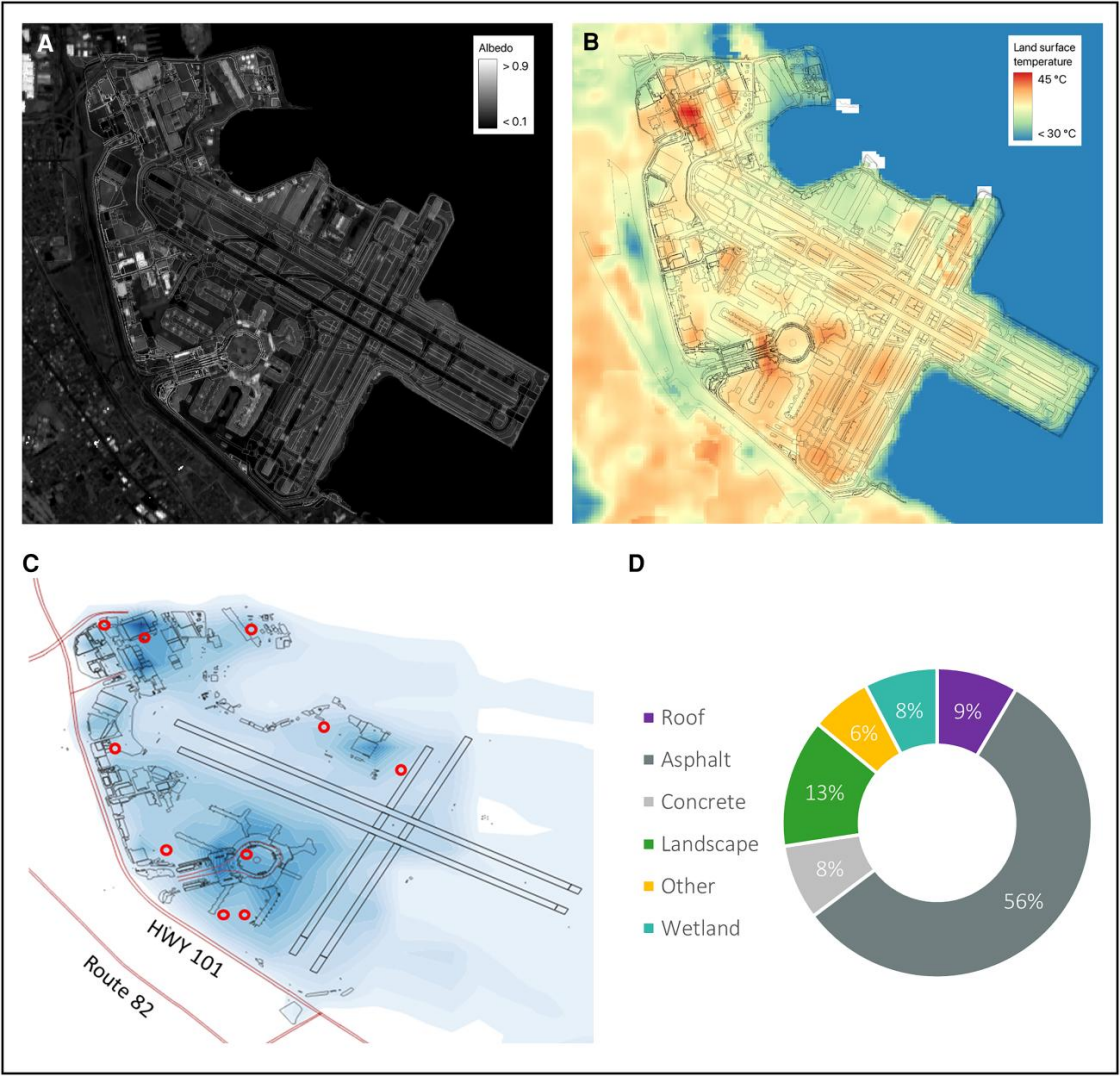
SFO airport-wide albedo, surface temperature and characteristics, and locations of interest. A) Albedo in 2023 at 10 m by 10 m resolution derived from Sentinel-2 satellite imagery, B) land surface temperature averaged for summer months in 2020–2023 at 30 m by 30 m resolution derived from 100-m by 100-m Landsat 8 satellite imaging, C) representative locations of interest (circles), as identified by airport stakeholders. The figure also shows an example reduction in air temperature from high albedo-increase scenario at SFO 1300 PDT on 2020 August 17 (darkest blue: −3.25 °C; lightest blue: −0.5 °C or smaller), and D) proportion of surface types at SFO based on existing geospatial land-use and land-cover data, described in the Supplemental Methods.

We identified 10 locations that would serve as representative points for our micrometeorological modeling across the airport’s diverse operational zones. To ensure that our selected outdoor locations would provide meaningful data, we focused on high-traffic areas where workers are frequently stationed or exposed to heat. We conducted a series of consultations with airport stakeholders who are knowledgeable about the daily activities of outdoor workers. Based on these discussions, the locations marked by circles in Fig. 1 were chosen.

### Surface characteristics analysis

Albedo was calculated using multiband data from the Sentinel-2 satellite (at 10 and 20 m resolutions) and Landsat 9 satellite (at 30 m resolution). First, the satellite data were filtered to contain only images for the area covering SFO and masked for clouds using the Cloud Score+ data product from Google with a threshold of 0.60. Albedo was calculated from the blue, green, and red visible (B2, B3, and B4), near-infrared (B8), and shortwave infrared (B11 and B12) bands using the narrow-to-broadband conversion coefficients as described in Bonafoni and Sekertekin (18). Details and code to calculate the surface characteristics in Google Earth Engine are provided in the Supplemental Methods. Albedo values range from 0 (indicating a nearly fully absorbing surface) to 1 (indicating a fully reflective surface). Some calculated values exceeded 1 due to factors such as sensor noise or highly reflective materials. Values >1 were set to a value of 1.

Three hypothetical albedo-increase scenarios were developed based on analysis of the SFO campus (Table 1). The baseline albedo of various surfaces (roofs, tarmacs, ramps, parking, streets, runways, blast pads, etc.) was each characterized at 10 and 30 m resolution. The three scenarios (low, moderate, and high) reflect incremental, realistic changes. The proposed interventions are technically feasible, and they draw on both existing technologies and examples of successful implementation elsewhere. A detailed example of surface change under the high albedo-increase scenario is provided in the Supplemental Material.

**Table 1.** Albedo values for baseline (current levels, i.e. “do nothing”) and three hypothetical scenarios of low, moderate, and high albedo modification for the micrometeorological modeling in SFO.

Surface type	Baseline (“do nothing”)	Hypothetical albedo modification scenarios		
		Low	Moderate	High
Roofs	0.28	0.63	0.75	0.85
Aprons	0.21	0.30	0.35	0.40
Parking	0.18	0.30	0.35	0.40
Roadways, shoulders, taxiways	0.18	0.30	0.35	0.40
Unclassified pavements	0.19	0.35	0.40	0.45
Runways	0.15	0.20	0.25	0.30

### Micrometeorological modeling

We used data inputs from multiple remote sensing platforms, atmospheric surface and upper air observations, as well as geometrical and urban morphological characterizations of the greater San Francisco Bay Area with a focus on SFO and its surroundings. The atmospheric and micrometeorological modeling done in this project was based on the highly urbanized Weather Research and Forecasting (WRF) and its Advanced Research WRF (ARW) modeling system (19). Model parameterizations were further modified and updated (12, 20, 21) to allow the simulations to go down to much higher resolutions, e.g. 50 m. The urban model was also modified to allow for multiple (different) parameterizations to run simultaneously to increase site specificity and accuracy of the model predictions. The atmospheric model was focused on summer conditions when heat mitigation from increased surface reflectivity is most effective. Several years and summer seasons were modeled. The analysis in this paper is restricted to August 2020 as an example of hottest conditions experienced at SFO.

In this study, we calculated outdoor wet bulb globe temperature (WBGT) accounting for air temperature (*T*_a_), wet bulb temperature (*T*_w_), globe temperature (*T*_g_), and wind speed (*u*) (22, 23). For conditions with no sunlight (shade or night):


(1) $$\mathrm{if}\;u>3\;m\;s^{-1};\mathrm{WBG}T_{\mathrm{no}\;\mathrm{sun}}=0.7\;T_{w}+0.3\;T_{a}$$

(2) $$\mathrm{if}\;u\leq 3\;m\;s^{-1};\;\;\;\mathrm{WBG}T_{\mathrm{no}\;\mathrm{sun}}=0.67\;T_{w}+0.33\;T_{a}-0.048\;\mathrm{lo}g_{10}\;u(T_{a}-T_{w})$$

For conditions with sun exposure (clear or overcast), the calculations followed the main expressions for WBGT:


(3) $$\mathrm{WBG}T_{\mathrm{sun}}=0.7\;T_{w}+0.2\;T_{g}+0.1\;T_{a}$$

where *T*_a_ is the postprocessed air temperature from the output of high-resolution micrometeorological model (modified WRF–ARW); *T*_w_ is calculated from Eq. 4 based on relative humidity (RH) and *T*_a_; and *T*_g_ based on Liljegren et al. (24) where Eq. 5 is solved iteratively, until |*T*_g_ new − *T*_g_ old| < 0.02 °C:


(4) $$\begin{array}{rl} & T_{w}=T_{a}\;\mathrm{ta}n^{-1}\{0.151977{\left(\mathrm{RH}+8.313659\right)}^{0.5}\}+\mathrm{ta}n^{-1}(T_{a}+\mathrm{RH})\\ & \;-\mathrm{ta}n^{-1}(\mathrm{RH}-1.676331)+0.00391838\;RH^{1.5}\;\mathrm{ta}n^{-1}(0.023101\;\mathrm{RH})\\ & \;-4.686035 \end{array}$$

(5) $$\begin{array}{r} T_{g}^{4}=\frac{L↓+L↑}{2\sigma }-\frac{h_{c}(T_{g}-T_{a})}{\varepsilon_{g}\sigma }+\frac{K↓(1-\alpha_{g})}{2\varepsilon_{g}\sigma }\left(1-F+\frac{F}{2\cos\;Z}\right)+\frac{1-\alpha_{g}}{2\varepsilon_{g}\;\sigma }K↑ \end{array}$$

Here, *L* is the longwave radiation, *h*_c_ is the convective heat transfer coefficient, *s* is the Stefan–Boltzmann constant, *e*_g_ is the emissivity of the globe, *a*_g_ is the albedo of the globe, *Z* is the solar zenith angle, *K* is the shortwave radiation, and *F* is the fraction of direct beam from total horizontal solar radiation.

### Productivity loss

Models that estimate worker’s productivity in challenging environments, like extreme heat, come from human experiments (chamber studies) that measure the body’s capacity to generate work under controlled conditions. Foster et al. (4) used a “fixed heart rate protocol” to measure the amount of work the body can generate at a fixed, maximally acceptable cardiovascular strain (130 beats per minute) across a broad spectrum of WBGT (range from 12 to 40 °C). The difference in cumulative work output, measured as total energy generated/expenditure in kilojoules (kJ), between cool and hot environmental conditions, is calculated to estimate reductions in physical work capacity, assuming workers are: (i) unacclimatized, (ii) performing moderate to heavy physical activities, (iii) dressed in normal work attire, and (iv) otherwise fit and healthy. Equation (6) is used to calculate the work loss for a given WBGT in a given working hour *i* (4), measured in minutes of work lost per hour per worker:


(6) $$\mathrm{Work}\;\mathrm{los}s_{i}=60-\left(\frac{1}{1+{\left(\frac{33.63}{\mathrm{WBG}T_{i}}\right)}^{-6.33}}\right)*\;60$$

We aggregated shifts and locations to compute the total work loss across all hours worked. Productivity percentages were calculated by comparing the work loss to the total potential work time, yielding measures for the percent productivity worked and lost at baseline conditions (do nothing), and the other three hypothetical albedo scenarios. After calculating productivity for each hour within a scenario, area under the curve was calculated by summing the areas of trapezoids formed between consecutive productivity measurements. By summing these individual areas, we obtained a measure of the cumulative productivity over time for each scenario.

## Results

Surface characteristics as well as albedo and surface temperature are described in Fig. 1. Across the area of SFO, land surface albedo measurements ranged from 0.08 over gravel to 0.85 over a single roof on the west side of the campus, with a low average campus albedo of 0.20 (Fig. 1A). Consistent with patterns of low albedo, temperatures in SFO were higher in areas in the west and south, as visualized in Fig. 1B. The urban heat island at SFO depends heavily on wind direction but is generally in the range of 1.4–2.2 °C (ambient temperature) higher than surroundings. Ambient temperature reduction from albedo change differs spatially across airport outdoor locations (Fig. 1C). When aggregating campus surfaces into six categories of interest (Fig. 1D), we found that almost two-thirds of SFO's surface area is made up of asphalt and concrete.

**Fig. 2.**
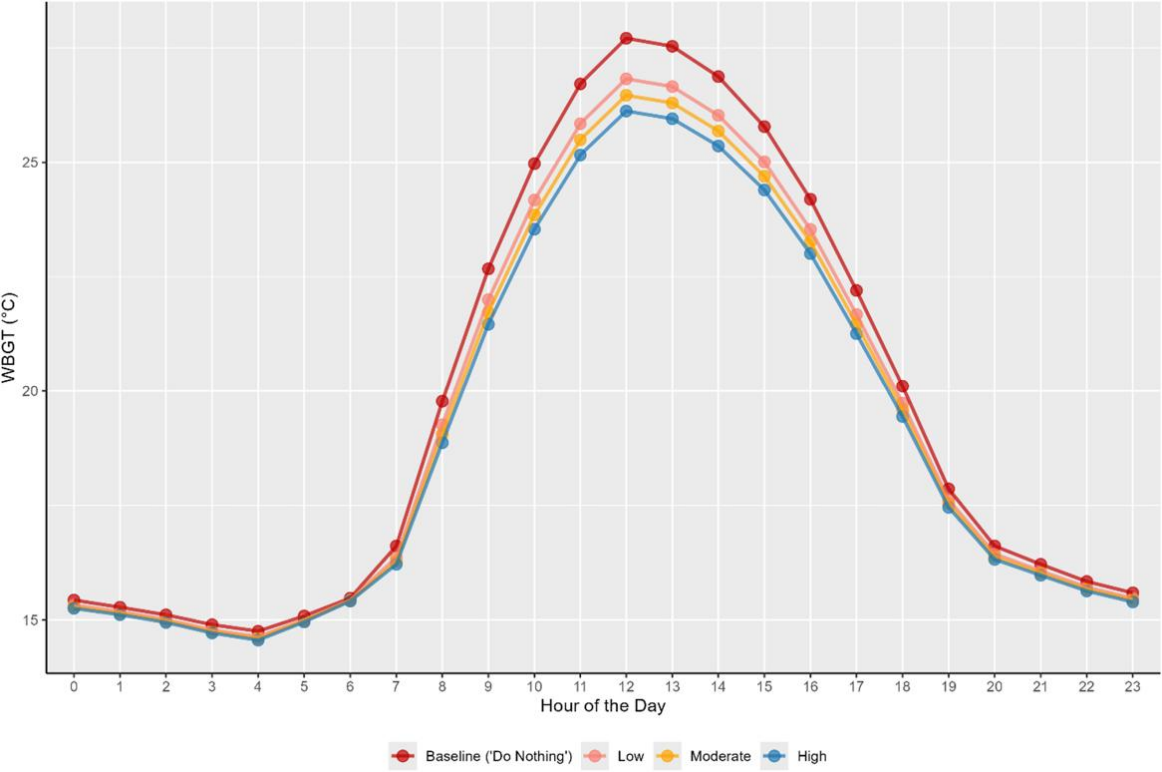
Average daily WBGT across the month of August 2020 in SFO under baseline conditions and the three hypothetical albedo modification scenarios.

When looking at WBGT, the data indicated a clear trend of temperature reduction with increasing levels of hypothetical albedo modification (Fig. 2). The largest absolute temperature reduction from potential albedo modification come during peak daytime hours. During midday (12pm), the average August WBGT in SFO outdoor locations was 27.71 °C. Adopting low, moderate, and high albedo modifications for SFO would reduce peak temperatures by 0.89, 1.25, and 1.59 °C, respectively. Similarly, across all hours, the average WBGT would be 19.72 °C at baseline and 19.32, 19.17, and 19.02 °C for the same hypothetical scenarios.

When accounting across workers shifts, the most significant temperature reductions are observed during the 7 AM–3 PM shift, where the baseline temperature of 24.11 °C decreases progressively to 23.39, 23.11, and 22.83 °C under low, moderate, and high albedo modifications, respectively. This is primarily driven by the midday hours (11 AM–3 PM) when solar radiation and surface heating are most intense, contributing significantly to WBGT peaks (Supplemental Table S1). The second shift (3 PM–11 PM) also shows some cooling but the temperature reductions are smaller than those observed during the first shift. The third shift (11 PM–7 AM) exhibits the smallest temperature reductions (Table 2). Heat retention in surfaces had a limited effect on WBGT during the evening and night hours when solar input decreases.

**Table 2.** WBGT values averaged across work shifts for baseline and three hypothetical scenarios of low, moderate, and high albedo modification in SFO in the month of August 2020.

Workers shifts	Baseline (“do nothing”) (°C)	Hypothetical albedo modification scenarios		
		Low (°C)	Moderate (°C)	High (°C)
First shift (7 AM–3 PM)	24.11	23.39	23.11	22.83
Second shift (3 PM–11 PM)	19.85	19.48	19.33	19.19
Third shift (11 PM–7 AM)	15.20	15.10	15.07	15.04
Total (24 h)	19.72	19.32	19.17	19.02

Compared with a baseline (“do nothing”) scenario, cool surfaces would have the largest impact on the productivity of workers during the first SFO shift of 7 AM to 3 PM. For workers in the first shift, there will be 5.20, 7.16, and 8.95 h gained per worker across the entire month in the low, moderate, and high albedo modification scenarios, respectively (Table 3). In total, high albedo modifications can provide up to 12 additional hours of physical work capacity per worker across all three shifts during the hottest months of the year.

**Table 3.** Hours gained per worker per shift per full summer month (August 2020), based on WBGT reductions resulting from each hypothetical albedo modification scenario (compared with baseline).

Workers shifts	Low vs. baseline (“do nothing”) (h)	Moderate vs. baseline (“do nothing”) (h)	High vs. baseline (“do nothing”) (h)
First shift (7 AM–3 PM)	5.20	7.16	8.95
Second shift (3 PM–11 PM)	1.75	2.40	3.00
Third shift (11 PM–7 AM)	0.06	0.08	0.10
Total^a^	7.01	9.64	12.05

^a^Total gained hours for one worker across all three shifts in a 24-h period.

## Discussion

In this study, we conducted a detailed and focused analysis of a busy airport with a large surface area, ideal for implementing design and engineering solutions. We assessed the impact of interventions such as cool surfaces on worker productivity. Our modeling exercise demonstrated that interventions aimed at mitigating workplace heat are beneficial not only for health but are also good for business. Specifically, we found that an aggressive change in surface albedo in SFO could increase productivity by an additional 12 h of physical work capacity per worker across all three shifts during the hottest months of the year. Even a relatively small change in albedo could result in up to 5.2 additional hours of productivity per worker per month in just one shift. These findings highlight the significant potential of workplace modifications to enhance both employee's well-being and employer's operational efficiency.

SFO, like other airports and urban areas, has extensive dark, impervious surfaces that exacerbate the urban heat island effect, making it an ideal site for implementing climate-resilient interventions. Increasing albedo across the campus, through measures such as cool roofs and pavements, is feasible and aligns with California’s building codes, which encourage reflective surfaces on new and replacement roofs. While vegetation is limited due to bird collision risks (25), we identified safe opportunities to expand greenery in nonoperational areas while maintaining aviation functionality (Supplemental Material). Beyond benefiting workers, optimizing how this airport manages sunlight and rainfall can significantly enhance infrastructure resilience, support energy and climate goals, and improve the health and well-being of both airport and airline workers and passengers. Smart surface solutions can provide a cost-effective method to integrate climate mitigation and adaptation into the airport’s growth. This airport, and many others, will inevitably face increasing heat challenges due to climate change.

The OSHA currently has no specific standards for regulating heat at the workplace, though it emphasized the urgency of this issue with an Advanced Notice of Proposed Rulemaking in 2021 (26) and a Notice of Proposed Rulemaking in 2024. Cal/OSHA has its own regulations for heat and outdoor workers under T8CCR3395, which includes provisions for rest, water, and shade (9). Cal/OSHA requires employers to take active measures at specific heat thresholds of 80 and 95°F (dry bulb; 26.7 and 35 °C). During the hot summer months at SFO, 80°F temperatures are often exceeded, whereas 95°F temperatures are not commonly exceeded (Supplemental Material). While these measures are crucial, employers at SFO will face challenges in maintaining target levels of heat comfort such as provision of shaded areas and ensuring availability of rest breaks (27). Implementing resilient and adaptive solutions, such as cool surfaces, can complement existing regulations by reducing ambient temperatures (dry and wet bulb) and easing the implementation of other heat mitigation strategies. This integrated approach offers a more effective way to enhance worker productivity while ensuring regulatory compliance.

Inaction in the face of worsening climate conditions is costly, in terms of both financial impact and worker productivity (5, 28, 29). Though SFO, and the Bay Area more broadly, have a relatively mild climate, simple interventions, such as upgrading older roofs to meet the California cool roof requirement to an albedo of 0.63, offer a low-hanging fruit solution that can yield significant benefits. Our analysis suggests that even modest albedo changes (i.e. the “low” scenario) could result in a productivity gain of 5.2 h per worker per month (i.e. for those in the first shift during the hot month of August). Based on an analysis of airfield door access, we estimate that there could be anywhere between 500 and 900 workers outdoors at any given time, especially during the first shift (analysis not shown). This could translate into thousands of additional productive hours, corresponding to substantial financial gains. Unfortunately, accurate numbers of outdoor workers at any given time were not available.

While intervention studies specifically focused on productivity gains from engineering solutions are limited (1, 29), insights from other fields offer valuable comparisons. For example, Prince et al. (30) demonstrated a 22% return on investment for a mix of administrative and engineering interventions like water, rest, and shade in agricultural settings in Nicaragua. Globally, a modeling study by Parsons et al. (5) found that with administrative controls of simply rescheduling labor to cooler parts of the day, up to 30% of global productivity losses from heat could be recovered. While our study focuses on engineering solutions, such as albedo modifications, these findings suggest that combining engineering and administrative strategies could further amplify the benefits of creating a safer and more productive environment for outdoor workers. Meanwhile, integrating safety practices with these interventions can ensure that the gains in productivity are achieved without compromising worker health (31).

This work has a number of limitations. First, the period of analysis was limited to a single summer month and did not provide year-long results. However, we chose the hottest month, which likely represents the period with the largest potential benefits. Second, this is a modeling study that relies on deterministic approaches. Such methods require assumptions that may not fully capture the complexity of real-world conditions. Some of these assumptions, such as estimated albedo changes and constant worker productivity responses, may not hold in all scenarios. Third, we recognize that the assumptions used in calculating productivity loss (such as workers being engaged in moderate to heavy physical activities, wearing standard work attire, and being fit and healthy) may not apply to all airport workers. Variability in worker tasks, physical effort, and exposure to heat across different airport locations could influence individual responses to heat and, therefore, the validity of our productivity estimates. For example, because acclimatization is rarely quantified, the productivity gains were modeled for unacclimatized workers, which may overestimate the benefits of cooling. Conversely, we assumed that all workers are fit and healthy, which may underestimate the benefits. On balance, these assumptions might offset each other, but this still introduces uncertainty. Future studies should account for these variations between workers to better reflect the diverse conditions faced by different worker groups. Fourth, other heat stress metrics should be considered. Different metrics, such as those accounting for sweating rates or clothing insulation, could provide a more comprehensive understanding of heat stress (32). Fifth, more field observations are needed to verify the results presented here. Future work should include a detailed cost–benefit analysis and consider longitudinal data to validate and refine these findings.

## Conclusion

We did a detailed modeling analysis for a large, bustling airport and demonstrated that climate-resilient interventions in the workplace, such as cool surfaces, may benefit both employees and employers. The potential benefits of these interventions include lowering temperatures and significantly increasing worker productivity. The results of this study can inform current and future design and construction projects.

## Supplementary Material

pgae593_Supplementary_Data

## Data Availability

A subset of the data (Dataset S1) is included in the Supplemental Material of this paper. The full dataset is available from the corresponding author (A.B.) upon reasonable request.
