# Supplementary material for: A modeling study of cool surfaces and outdoor workers productivity at San Francisco International Airport
Source: PNAS Nexus. 2025 Jan 3;4(1):pgae593. doi: 10.1093/pnasnexus/pgae593 (PMC11740725; doi:10.1093/pnasnexus/pgae593)
Supplement: pgae593_Supplementary_Data [file pgae593_supplementary_data.zip › PNASNEXUS-PNASNEXUS-2024-01157R-s01.docx]

**SUPPLEMENTAL MATERIAL**

1. Supplemental Methods
   1. Albedo
   2. Fractional Vegetation
   3. Land Surface Temperature
   4. Code
   5. Model Validation
2. Supplemental Tables
   1. Table S1. Wet bulb globe temperature (WBGT) in the three hypothetical scenarios
3. Supplemental Figures
   1. Figure S1. Modeled vs. observed air temperature at SFO weather station
   2. Figure S2. Example changes of surfaces under the high albedo increase scenario
   3. Figure S3. Advected cool plumes across the SFO campus
   4. Figure S4. Ambient temperature (in °F) reduction under albedo change scenarios
   5. Figure S5. Fractional vegetation across the SFO campus
4. Supplemental Dataset
   1. Dataset S1. Hourly wet bulb globe temperature (WBGT) in the three hypothetical scenarios averaged across all locations
5. ***Supplemental Methods***
   1. ***Albedo***

*Albedo and fractional vegetation are both calculated using images from the Sentinel-2 satellite using Google Earth Engine. First, the satellite data is filtered to contain only images for the year 2023 for the area covering SFO and masked for clouds using the Cloud Score+ data product from Google with a threshold of 0.60.*

*Albedo (𝛼 ) was calculated from the blue, green, and red visible (B2, B3, B4), near infrared (NIR, B8), and shortwave infrared (SWIR, B11, B12) bands using the narrow-to-broadband conversion coefficients presented in* [*Bonafoni and Sekertekin (2020):*](https://ieeexplore.ieee.org/document/8974188)

*𝛼 = 𝐵2 × 0.2266 + 𝐵3 × 0.1236 + 𝐵4 × 0.1573 + 𝐵8 × 0.3417 + 𝐵11 × 0.1170 + 𝐵12 × 0.0338*

*Values greater than 1 were set to a value of 1. In this process the SWIR bands are downscaled to match the spatial resolution of 10-m. The albedo estimate image shows the pixel-wise means for 2023 at a spatial resolution of 10-m.*

- 1. ***Fractional vegetation***

*The fractional vegetation (representing the fraction of a pixel covered by green vegetation) is calculated from the normalized difference vegetation index (NDVI) as presented in* [*Gillies and Carlson (1995)*](https://journals.ametsoc.org/view/journals/apme/34/4/1520-0450_1995_034_0745_trsoss_2_0_co_2.xml?tab_body=abstract-display)*. First, the NDVI is calculated as a ratio between the red and NIR bands:*

$$NDVI=\frac{B8-B4}{B8+B4}$$

*Fractional vegetation was calculated from the maximum NDVI values for 2023 as:*

$$Fr=\left( \frac{NDVI-NDVIsoil}{NDVIveg-NDVIsoil} \right)^{2}$$

*with 𝑁𝐷𝑉𝐼𝑠𝑜𝑖𝑙 = 0.1 and 𝑁𝐷𝑉𝐼𝑣𝑒𝑔 = 0.7 being chosen from manual selection of pixels representing bare earth and full vegetation. Values greater than 1 were set to a value of 1. The fractional vegetation image shows the pixel-wise fractional vegetation derived from the*

*maximum NDVI for 2023 at a spatial resolution of 10-m.*

- 1. ***Land Surface Temperature***

*Land surface temperature (LST) was calculated from the Landsat 8 satellite and was processed using Google Earth Engine. First, the satellite data is filtered to contain only images for the June, July, and August 2020-2023 for the area covering SFO and with less than 20% cloud cover. Cloudy pixels were also masked from the calculations using the QA band. Because the Landsat satellite has a revisit time of 8 days, multiple years were used to ensure that there were enough cloud-free pixels to estimate the mean temperature. The LST image shows the pixel-wise means for summer 2020-2023 at a spatial resolution of 30-m. However, it is important to note that the satellite records LST at a spatial resolution of 100-m but is downscaled to a resolution of 30-m to match the resolution of the other bands.*

- 1. ***Code***

*The code used to calculate the surface characteristics in Google Earth Engine can be found* [*here*](https://code.earthengine.google.com/f95a1035f3a176e3105010e547a04c16)*:* [*https://code.earthengine.google.com/f95a1035f3a176e3105010e547a04c16*](https://code.earthengine.google.com/f95a1035f3a176e3105010e547a04c16)

- 1. ***Model Validation***

*Model performance was evaluated according to the following criteria, as defined and discussed in Taha (2008). These criteria ensure that the model meets acceptable accuracy standards:*

- *Air Temperature: Gross error ≤ 2 K, Bias ≤ ±0.5 K, Index of Agreement (IOA) ≥ 0.8*
- *Humidity: Gross error ≤ 2 g kg⁻¹, Bias ≤ ±1 g kg⁻¹, IOA ≥ 0.6*
- *Wind Speed: Root Mean Square Error (RMSE) ≤ 2 m s⁻¹, Bias ≤ ±0.5 m s⁻¹, IOA ≥ 0.6*

*As an example, Figure S1 below compares the model's air temperature predictions with observed air temperature at the KSFO weather station, showing a bias of 0.27 °C and a gross error of 1.19 °C. It is important to note that the KSFO weather station is located by the water (San Francisco Bay), which is significantly cooler than areas closer to the built-up sections of the airport (see Figure S2 for station location).*

***References (supplemental methods):***

*Bonafoni S, Sekertekin A, 2020. Albedo Retrieval From Sentinel-2 by New Narrow-to-Broadband Conversion Coefficients. IEEE Geosci Remote Sensing Lett. Sep;17(9):1618–1622.*

*Gillies RR, Carlson TN, 1995. Thermal Remote Sensing of Surface Soil Water Content with Partial Vegetation Cover for Incorporation into Climate Models. J Appl Meteor Apr; 34(4) 745-756*

*Taha H, 2008. Episodic Performance and Sensitivity of the Urbanized MM5 (uMM5) to Perturbations in Surface Properties in Houston Texas. Boundary-Layer Meteorology, 127:193–218.*

1. ***Supplemental Tables***
   1. **Table S1.** Wet bulb globe temperature (WBGT) average and standard deviation for every hour in °C for baseline and three hypothetical scenarios of low, moderate, and high albedo modification in SFO in the month of August 2020.

| **Hour** | **Baseline**  **(‘Do Nothing’)** | **Hypothetical Albedo Modification Scenarios** | | |
| --- | --- | --- | --- | --- |
|  |  | **Low** | **Moderate** | **High** |
| 0 | 15.43 (1.01) | 15.32 (1.01) | 15.28 (1.02) | 15.25 (1.03) |
| 1 | 15.28 (0.99) | 15.18 (0.99) | 15.14 (1.00) | 15.11 (1.01) |
| 2 | 15.11 (0.98) | 15.01 (0.98) | 14.97 (0.99) | 14.94 (0.99) |
| 3 | 14.89 (0.99) | 14.78 (1.01) | 14.75 (1.02) | 14.71 (1.02) |
| 4 | 14.75 (1.00) | 14.63 (1.02) | 14.60 (1.02) | 14.56 (1.02) |
| 5 | 15.09 (1.08) | 15.00 (1.09) | 14.98 (1.10) | 14.95 (1.11) |
| 6 | 15.47 (1.15) | 15.43 (1.17) | 15.42 (1.17) | 15.41 (1.17) |
| 7 | 16.61 (0.97) | 16.36 (1.02) | 16.28 (1.04) | 16.21 (1.07) |
| 8 | 19.78 (1.41) | 19.26 (1.34) | 19.06 (1.32) | 18.87 (1.30) |
| 9 | 22.67 (1.64) | 22.00 (1.57) | 21.73 (1.55) | 21.46 (1.53) |
| 10 | 24.97 (1.68) | 24.18 (1.64) | 23.85 (1.63) | 23.54 (1.62) |
| 11 | 26.71 (1.90) | 25.84 (1.87) | 25.49 (1.86) | 25.16 (1.86) |
| 12 | 27.71 (2.24) | 26.82 (2.25) | 26.46 (2.26) | 26.12 (2.27) |
| 13 | 27.53 (1.93) | 26.65 (1.97) | 26.30 (1.99) | 25.95 (2.01) |
| 14 | 26.87 (1.70) | 26.03 (1.73) | 25.68 (1.76) | 25.36 (1.78) |
| 15 | 25.78 (1.60) | 25.01 (1.64) | 24.70 (1.66) | 24.40 (1.68) |
| 16 | 24.19 (1.52) | 23.53 (1.53) | 23.27 (1.54) | 23.01 (1.56) |
| 17 | 22.20 (1.41) | 21.68 (1.41) | 21.46 (1.42) | 21.26 (1.42) |
| 18 | 20.11 (1.27) | 19.74 (1.27) | 19.59 (1.27) | 19.44 (1.27) |
| 19 | 17.86 (1.09) | 17.63 (1.10) | 17.54 (1.11) | 17.46 (1.12) |
| 20 | 16.61 (1.05) | 16.44 (1.07) | 16.38 (1.09) | 16.32 (1.10) |
| 21 | 16.21 (1.07) | 16.07 (1.10) | 16.02 (1.11) | 15.97 (1.12) |
| 22 | 15.84 (1.07) | 15.72 (1.09) | 15.67 (1.10) | 15.63 (1.11) |
| 23 | 15.59 (1.03) | 15.47 (1.04) | 15.43 (1.05) | 15.39 (1.06) |

1. ***Supplemental Figures***


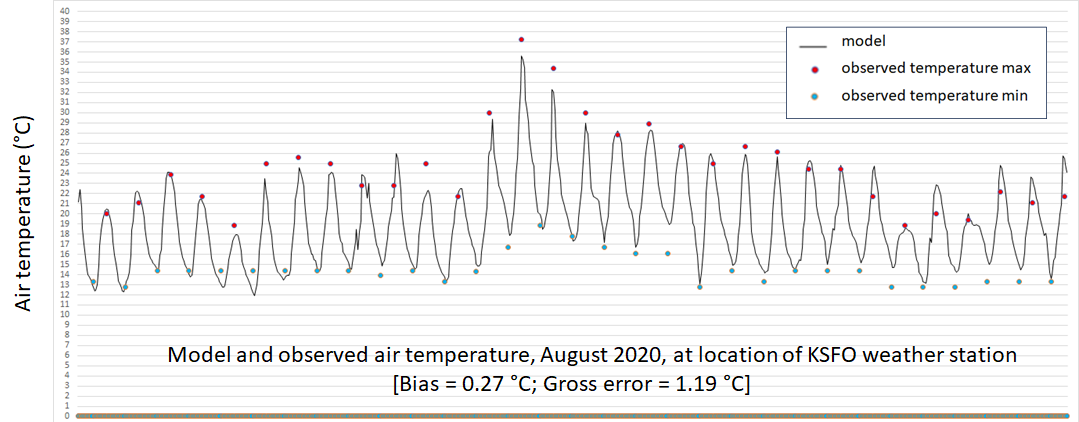


**Figure S1**. Modeled vs. observed air temperature at KSFO weather station for August 2020, showing a bias of 0.27°C and a gross error of 1.19°C.


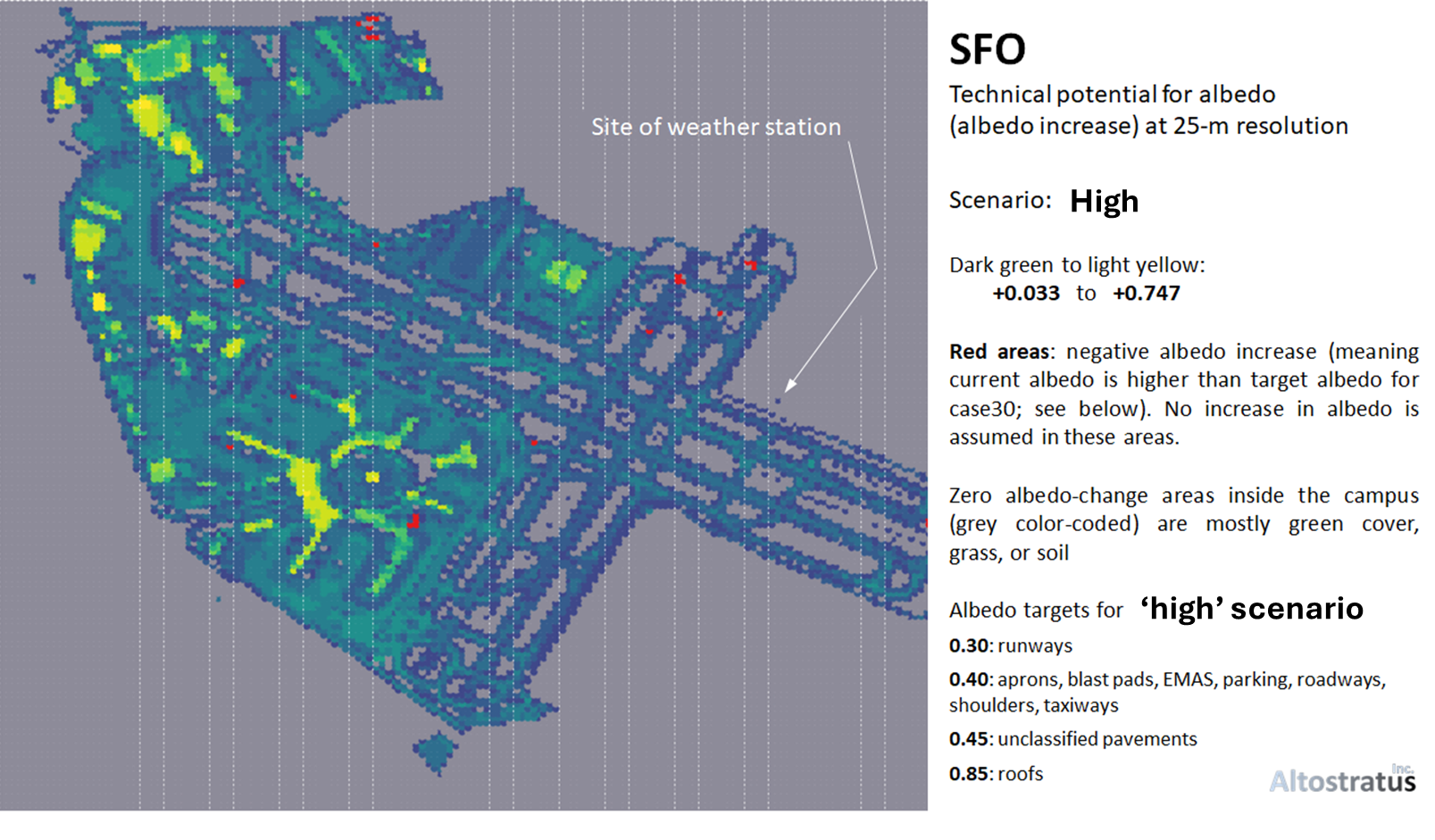


**Figure S2.** Example changes of surfaces in SFO under the high albedo increase scenario


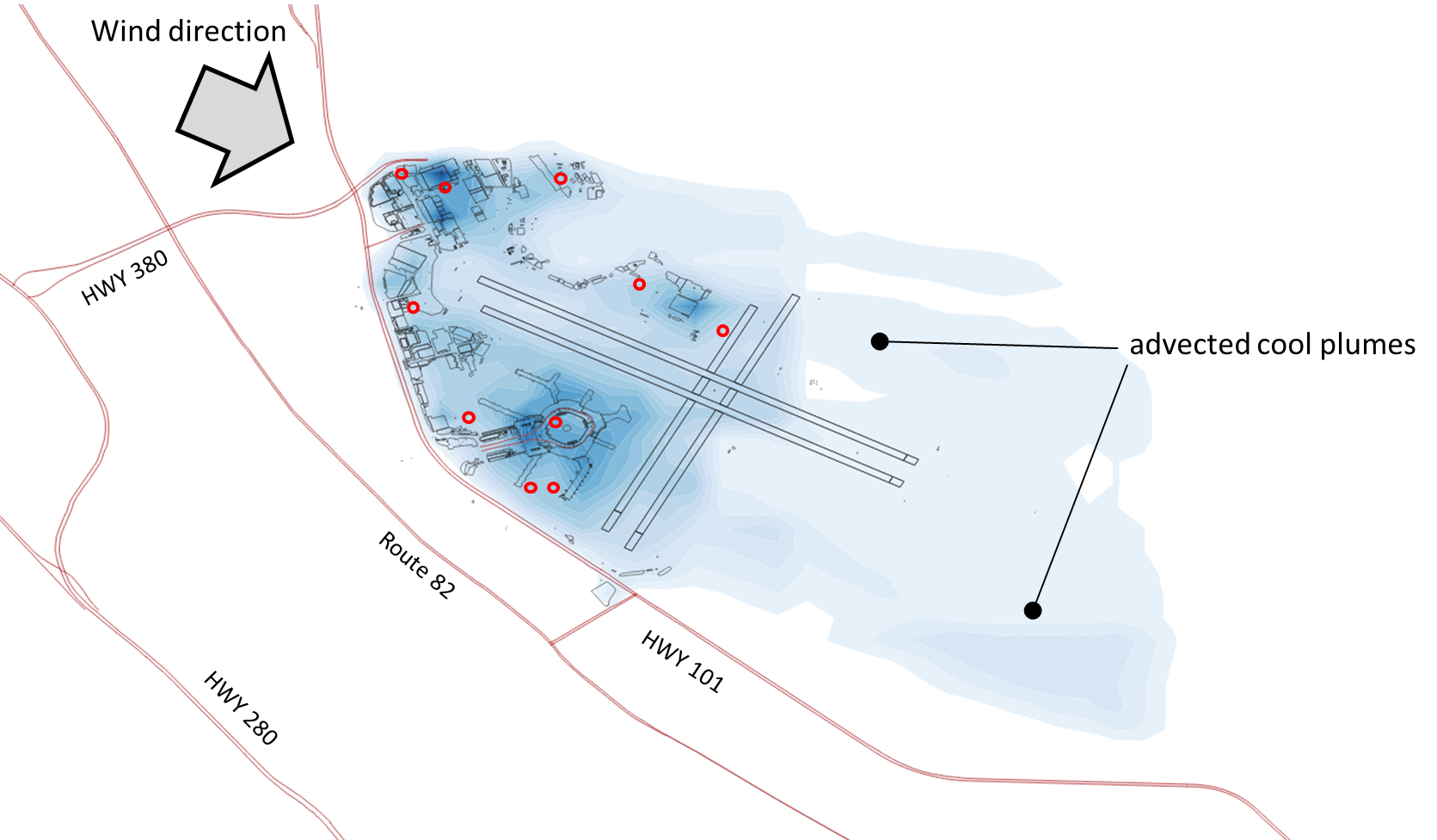


**Figure S3.** Reduction in 2-meter air temperature from the high albedo increase scenario at SFO on August 17^th^, 2020 at 1PM PDT. Results are from a modified version of the urban WRF-ARW model at resolutions of 100 – 50 m. Darkest blue: -3.25 °C; lightest blue: -0.5 °C or smaller. Red circles represent sampling points for micrometeorological variables. The results demonstrate an advected cooling plume generated by the prevailing wind direction, which cools areas beyond the primary high-traffic zones.

Maximum Temperature:

**
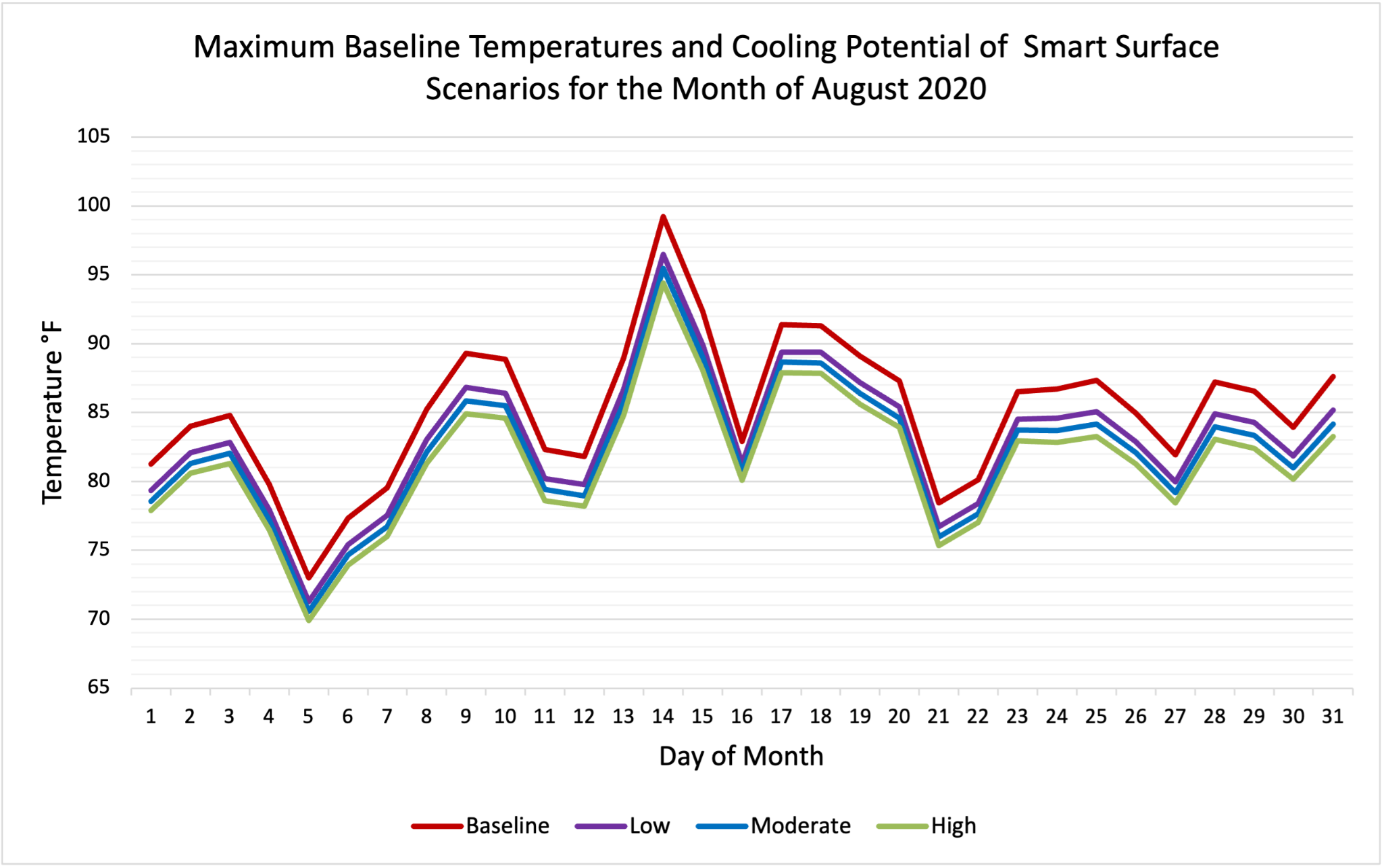
**

Average Temperature:

**
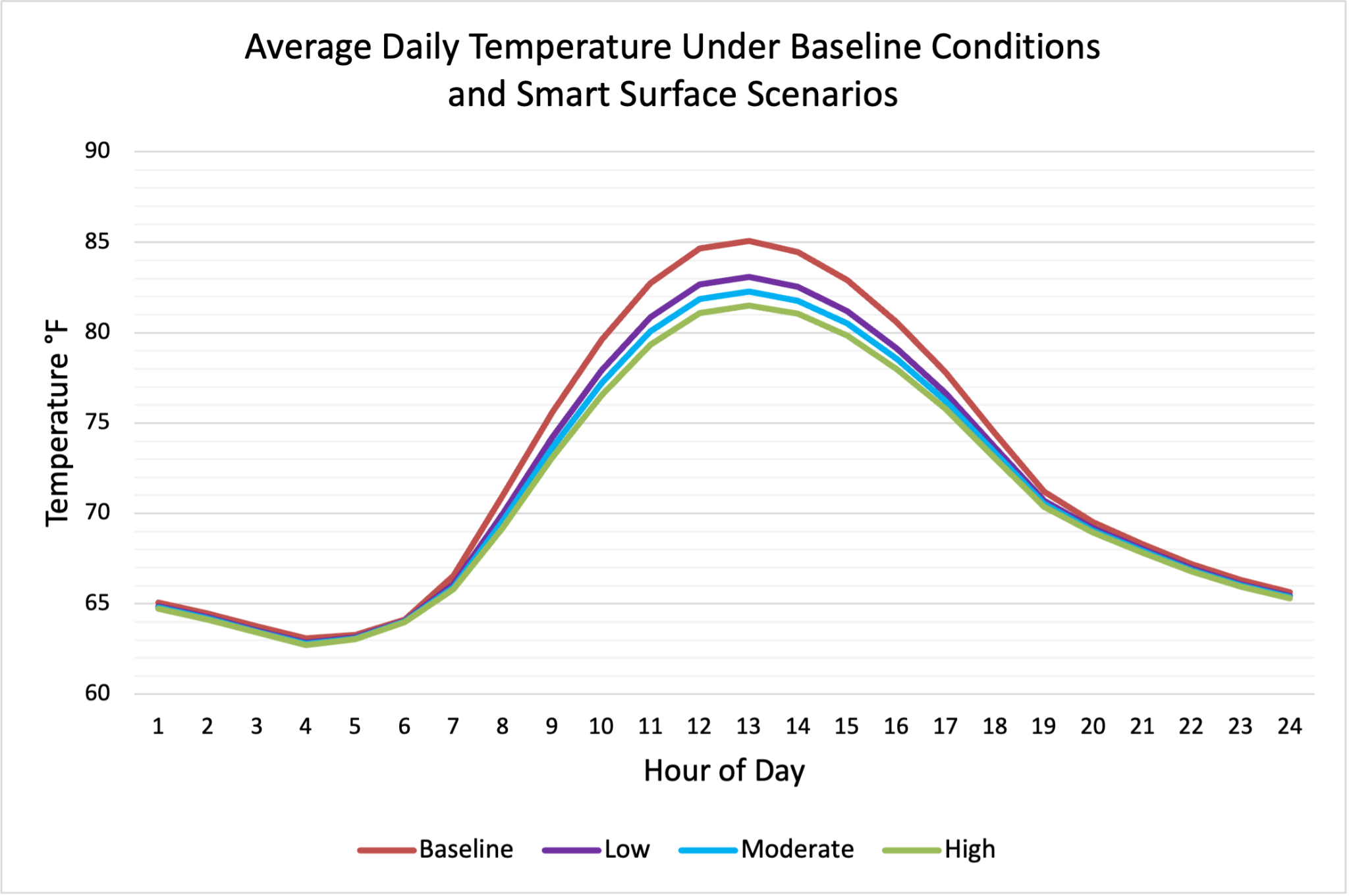
**

**Figure S4**. Temperature distribution across the month of August 2020 under baseline conditions and three albedo modification scenarios


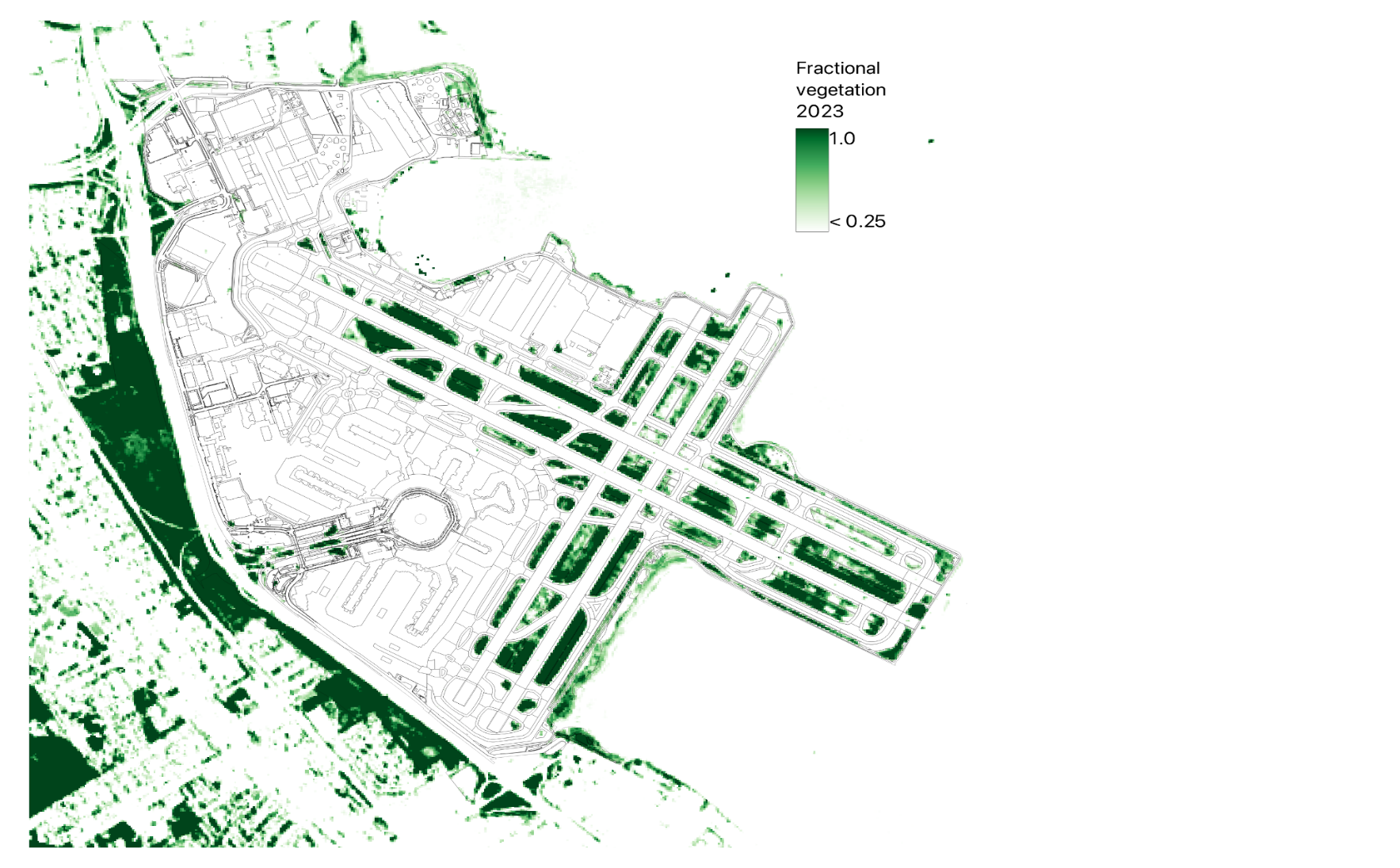


**Figure S5.** SFO fractional vegetation cover calculated from 10-meter Sentinel-2 satellite imagery from 2023. Values range from 0 over much of the airport to 1 over vegetated areas of the West-of-Bayshore wetland. The analysis reveals that vegetation is primarily limited to the West-of-Bayshore wetland area and grassy runway medians. Expanding vegetation in airside areas is limited due to the risk of wildlife collisions. However, we identified opportunities to safely expand vegetation on the landside areas of the campus, away from active aircraft operations, which could contribute to cooling without impacting aviation functionality.
